# The Dark Side of Microalgae Biotechnology: A Heterotrophic Biorefinery Platform Directed to ω-3 Rich Lipid Production

**DOI:** 10.3390/microorganisms7120670

**Published:** 2019-12-10

**Authors:** Teresa Lopes da Silva, Patrícia Moniz, Carla Silva, Alberto Reis

**Affiliations:** 1 LNEG-National Laboratory for the Energy and Geology, I.P.-Bioenergy Unit; Estrada do Paço do Lumiar, 22, Edifício F–R/C, 1649-038 Lisboa, Portugal; patricia.moniz@lneg.pt (P.M.); alberto.reis@lneg.pt (A.R.); 2 Instituto Dom Luiz (IDL), Faculdade de Ciências da Universidade de Lisboa, Campo Grande, 1747-016 Lisboa, Portugal; camsilva@fc.ul.pt

**Keywords:** heterotrophic microalgae, low-cost substrates, circular economy, eicosapentaenoic acid (EPA), docosahexaenoic acid (DHA), bioenergy, biodiesel, sustainability indicators

## Abstract

Microbial oils have been considered a renewable feedstock for bioenergy not competing with food crops for arable land, freshwater and biodiverse natural landscapes. Microalgal oils may also have other purposes (niche markets) besides biofuels production such as pharmaceutical, nutraceutical, cosmetic and food industries. The polyunsaturated fatty acids (PUFAs) obtained from oleaginous microalgae show benefits over other PUFAs sources such as fish oils, being odorless, and non-dependent on fish stocks. Heterotrophic microalgae can use low-cost substrates such as organic wastes/residues containing carbon, simultaneously producing PUFAs together with other lipids that can be further converted into bioenergy, for combined heat and power (CHP), or liquid biofuels, to be integrated in the transportation system. This review analyses the different strategies that have been recently used to cultivate and further process heterotrophic microalgae for lipids, with emphasis on omega-3 rich compounds. It also highlights the importance of studying an integrated process approach based on the use of low-cost substrates associated to the microalgal biomass biorefinery, identifying the best sustainability methodology to be applied to the whole integrated system.

## 1. Introduction

Microalgae produce a wide range of valuable compounds such as carbohydrates, proteins, pigments and lipids. Currently, they are considered potential contributors to the world’s bio-economy development due to their capacity of producing marketable high-value added products from low-value products as feedstock. Moreover, microalgae can grow on low-cost substrates such as industrial byproducts or effluents, resulting in pollutant removal with concomitant production of biocompounds with commercial interest. The non-seasonality, the non-dependence on climatic conditions and the non-need of arable land to place and operate microalgae bioreactors is a major strength of this feedstock.

Oleaginous microalgae produce intracellular oils above 20% of their dry weight that can be converted into biodiesel. However, biodiesel obtained from microbes, including microalgae, is still not economically sustainable since its production price is still not competitive compared with fossil fuel price (the estimated cost for biodiesel produced from a heterotrophic microorganism is 4.8 times the price for conventional diesel [1]). One way to reduce the costs consists of using low-cost substrates in the culture medium, namely industrial/domestic effluents, byproducts and residues. Another approach, based on the biorefinery concept applied to microalgae-based bioprocesses, takes advantage of the various products synthesized by the microalgae, maximizing the value derived from the whole process, aiming at a desired zero waste. In this way, the economics of the full process may be greatly improved, as the high added valued biocompounds such as PUFAs, carotenoids, proteins, etc., may contribute to sustain the microbial biofuel production [2].

PUFAs have an important role in membrane fluidity, cellular metabolism, transport, and as eicosanoid. The benefits of ω-3 PUFAs on human health are well known, which have attracted the interest of pharmaceutical, nutraceutical, cosmetic, food and feed industries. ω-3 PUFAs are important brain and nervous system components, playing a crucial role in several neurological functions, such as neurogenesis, neurotransmission, and protection against oxidative stress-induced cerebral damage.

The precursor of ω-3 PUFAs is alfa linolenic acid (18:3ω3) which can be converted into eicosapentaenoic acid (EPA, 20:5ω3), docosapentaenoic acid (DPA, 22:5ω3) and docosahexaenoic acid (DHA, 22:6ω3), but the conversion rate is very low. Consequently, ω-3 PUFAs must be taken up through the diet [3]. Particularly long-chain DHA and EPA play an important role in the treatment of many human diseases such as cancer, atherosclerosis, rheumatoid arthritis, Alzheimer’s, and psoriasis. DHA is an essential component of the brain and nervous system, being a crucial fatty acid among the ω-3 PUFAs as it plays an important role in the development of an infant’s brain. DHA has become more popular following research reports that claim that many formula-fed infants have lower levels of DHA and arachidonic acid (ARA, 20:4ω6) as compared to breast-fed infants. This fact explains the global microalgae-based DHA market increase, attributed to the rising public awareness about healthcare and chronic diseases, which has led to the expansion of the DHA applications and regulations, favoring the product usage in infant formulations [4,5].

The main sources of ω-3 PUFAs, including DHA and EPA, are fatty fish species, such as herring, mackerel, sardine, menhaden and salmon [6]. However, the global fish stocks are declining and cannot provide a sustainable source of ω-3 fatty acids. On the other hand, the quality of fish oil is variable and depends on fish species, season and location of catching sites. In addition, fish oils present an unpleasant smell and may be contaminated by polychlorobiphenyls (PCBs) and heavy metals, making them inappropriate to be incorporated in food and feed, namely in infant formulas, or to be used in pharmaceutical formulations. Furthermore, as marine fish oil is a complex mixture of fatty acids with varying lengths and degrees of unsaturation, expensive DHA purification may be required before application. Oils from genetically modified plants and microorganisms are two potential alternatives to fish oil (even though ω-3 PUFAs contents can be higher in the latter). Although transgenic plants present numerous advantages, their production are dependent on seasonal and climatic conditions as well as on the availability of arable land. Moreover, there are public concerns regarding the cultivation of transgenic crops in open ecosystems [3].

In this way, microalgae biomass is particularly suitable for the extraction and purification of individual PUFAs as it is cholesterol free, contaminant free (e.g., heavy metals, polychlorobiphenyls (PCBs)), and tastes good [7]. At present, algal oil represents less than 2% of human EPA/DHA consumption, but its contribution has been increasing due to several social attributes including its environmental friendliness, the absence of ocean borne contaminants, its vegetarian nature, as well as the possibility to be manufactured under kosher or halal conditions. However, microalgae-based oils from fermentation and refining are currently more expensive than fish oils. Thus, microalgae based production of EPA/DHA-enriched oils can accommodate greater demand for these nutrients, although at a higher price [5].

Autotrophic microalgae use carbon dioxide and light as carbon and energy sources, sequestering 1.83 kg CO_2_ kg^−1^ dry microalgae as average [8], while heterotrophic microalgae use organic carbon as a source of carbon and energy. It is quite challenging to find the CO_2_ emissions during the heterotrophic growth, but one can estimate 0.1–4 kg CO_2_ kg^−1^ dry microalgae (see Section 9). Autotrophic microalgae cultivations mitigate the greenhouse gas emissions (GHG) due to their CO_2_ consumption and O_2_ production, and may use non-potable water and non-arable land, which are advantages over heterotrophic cultivations. Cheaper phototrophic microalgae cultivation systems, such as open raceways, can be constructed on degraded, marginal and non-agricultural lands, preserving the use of high-value lands and crop producing areas. However, photoautotrophic cultures show several limitations: (i) continuous and clean water supply is required; (ii) poor light diffusion could occur in the culture, which is intensified with depth, being aggravated when cultivation is intensive causing self-shading, resulting in light limitation for the cells, which will inevitably lead to low biomass productivities; (iii) easy contamination, competition, infestation and predation by other organisms, being difficult to maintain pure microalgae cultures; (iv) dependence on season and weather conditions and (v) difficulty in microalgal biomass harvesting due to low biomass concentrations. To overcome these limitations, photobioreactors (PBR) may be used allowing microalgal cultivations to be developed under controlled conditions, being possible to maintain pure cultures aiming to produce pharmaceutical products. However, these systems also present some drawbacks such as high initial investment in the infrastructure, and continuous maintenance is required; when using large culture volumes, an efficient light dispersion through the culture is difficult to maintain, which is aggravated if biofilms are developed on the PBR surfaces, which will inevitably lead to light limitation conditions [9]. Moreover, autotrophic microalgae cultivations in PBR located at higher latitudes require heating systems and expensive greenhouse infrastructures to maintain high productivity, increasing the process costs. It is estimated a 33% autotrophic process yield penalty by moving to 40° N from the equator [10].

Therefore, heterotrophic microalgae may be particularly interesting for countries located at higher latitudes such as European countries. In addition, heterotrophic microalgae cultivations are conducted in conventional fermenters, under strictly axenic and operational conditions, being less prone to microbial contaminations than autotrophic cultures when conducted outdoors. Consequently, heterotrophic conditions can enhance the microalgal biomass concentration compared with phototrophic conditions [11,12], leading to higher biomass and lipids productivities [13]. In addition, in most cases, heterotrophic cultivation systems are cheaper, easier to maintain on large scale than autotrophic cultivation, and facilities are simpler to be constructed [9].

This review analyses the different strategies that have been recently used to cultivate heterotrophic microalgae for lipids, with emphasis on ω-3 compounds, emphasizing the importance of an integrated process approach based on the use of a multi-product microalgal biorefinery that uses efficiently all microalgae fractions, in order to achieve a sustainable process.

## 2. Heterotrophic Metabolism—Carbon Uptake and Lipid Synthesis

**Figure 1:**
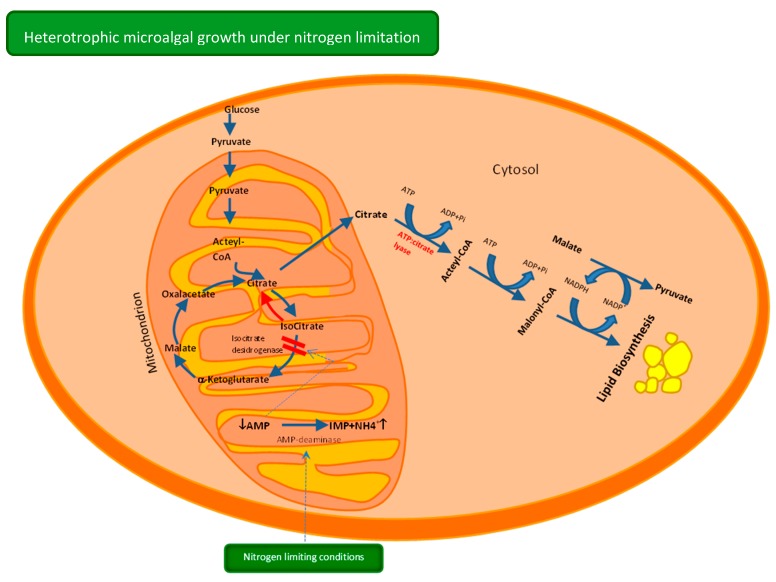
Carbon uptake and lipid synthesis in heterotrophic microalgae under nitrogen limiting conditions.

In heterotrophic metabolism, the organic carbon uptaken by microalgae is broken down in the same way as bacteria is. Organic carbon is consumed by heterotrophic microalgae for energy production. Complex molecules like starch can be metabolized via Embden-Mayerhoff-Parnas Pathway (EMP pathway or glycolysis) or the Pentose Phosphate pathway (PPP), generating NADH and ATP [14]. In the first pathway, starch is firstly broken down to glucose which is eventually phosphorylated and directed to the EMP pathway. The final product of this pathway, pyruvate, is formed in the cytosol, and results from the glyceraldehyde-3-phosphate conversion, previously exported into the cytosol. Generally, oleaginous organisms are AMP-dependent of isocitrate dehydrogenase, an enzyme of the TCA cycle which catalyses the isocitrate oxidative decarboxylation. Under nitrogen limiting conditions, the adenosine monophosphate (AMP) deaminase is converted into inosine monophosphate (IMP) and ammonia which is supplied to the nitrogen-starved cells causing the depletion of AMP levels. The isocitrate metabolism is blocked resulting in its accumulation and equilibrium with citrate, in the mitochondrion (Figure 1). The citrate excess is transported out of the mitochondrion to the cytosol. ATP:citrate lyase, a key enzyme in the oleagenicity, will cleave citrate in the cytosol, resulting in acetyl-CoA synthesis for fatty acids production [15]. This pathway almost ends in the palmitic (16:0) or stearic (18:0) acids synthesis. For long-chain fatty acids synthesis, including PUFAs synthesis, subsequent elongation and desaturation series involving elongases and desaturases are required. Only fungi and algae have the capacity to synthesize lipids containing more than 20% PUFAs (*w/w* total fatty acids), which make them attractive for this goal [15].

The ability of eukaryotic oleaginous organisms to accumulate a large amount of lipids is not different in terms of fatty acid biosynthesis compared to non-oleaginous species. However, a continuous supply of acetyl-CoA and NADPH for the fatty acid production by a reversed ω-oxidation has to be assured under nutrient limited but carbon excess conditions. Heterotrophic algae growing under aerobic conditions respire, which, as mentioned above, occurs with the complete oxidation of glucose to CO_2_ via EMP, PPP and the tricarboxylic acid cycle (TCA cycle), and ATP is generated by oxidative phosphorylation. Therefore, algal cultures grown on carbon sources, such as glucose, require an efficient aeration of the cultures to obtain high biomass productivities, since oxygen is required for respiration. When using heterotrophic microalgae for lipid production, in order to make the process economically and environmentally sustainable, it is important that the selected species (i) can grow on relatively inexpensive sterilized media; (ii) show ability to withstand hydrodynamic stresses that exist in conventional fermenters; (iii) show adaptability to harsh environmental conditions and (iv) show ability to utilize a variety of organic carbon sources including waste lignocellulosic biomass and other materials [14].

## 3. Oleaginous Heterotrophic Microalgae Strains for ω-3 Compounds

Table 1 shows the most used heterotrophic microalgae or microalgae-like strains that have been reported in literature to produce DHA and EPA, as well as low-cost carbon sources that have been used to produce ω-3 compounds. Currently, the most used microalgae for the production of ω-3 rich algal oil and biomass are marine members of the families Thraustochytriacea and Crythecodiniacea, which are present in the oceans. *Crypthecodinium* is a genus of the family Crypthecodiniaceae. The Thraustochytrids include the genera *Aurantiochytrium*, *Schizochytrium* and *Ulkenia*. These heterotrophs can display a high oil content (up to 50–77% on a dry weight basis) which is mainly composed of triacylglycerols (TAGs) rich in DHA.

## 4. Effect of Operational Conditions on Microalgal Growth, Lipids and DHA Production

### 4.1. Medium Composition

The carbon source is the most expensive component of the fermentation media. In the late 1990s and early 2000s, single carbon substrates such as glucose, ethanol, acetate and glycerol have been used to grow heterotrophic microalgae for ω-3 compounds [16,17,18,19,20,21,22,23,24]. studied *C. cohnii* 30772 batch growth on medium containing 25 g L^−1^, 50 g L^−1^ and 75 g L^−1^ of glucose and found that maximum biomass concentration was attained at the highest glucose concentration, although the specific growth rate decreased for glucose concentrations higher than 25 g L^−1^. It is important to consider this inhibitory effect of high glucose concentrations on *C. cohnii* growth when growing these microalgae in fed-batch systems, in order to avoid substrate inhibition.

However, despite these carbon sources inducing high lipid and DHA productivities, they are expensive (glucose 16 € kg^−1^; ethanol 1.82 € kg^−1^; acetic acid 0.45 € kg^−1^, www.alibaba.com), being ethanol and acetic acid are dangerous and difficult to handle and transport. Other expensive components of the medium culture are nitrogen compounds. Inorganic nitrogen sources (e.g., ammonia, urea) can be used, but they lack the trace minerals and other nutrients (i.e., vitamins) which are essential to micro-algal growth and are present in complex nitrogen sources such as degraded proteins as yeast extract and soya peptones. However these nutrients are expensive (yeast extract: 35.4 € kg^−1^; soya peptones: 7.25 € kg^−1^, www.alibaba.com). The increasing public awareness of the need to accomplish the circular economy rules, as well as the need to use low-cost substrates as feedstock to reduce bioprocess costs, have led to search wastes/byproducts/effluents to be used as nutrients in media formulations for microbial growth. In fact, in recent years, substrates as food waste hydrolysates, sweet sorghum juice, carob pulp syrup, rapeseed meal hydrolysate mixed with crude molasses (0.91 € kg^−1^, www.alibaba.com), cheese whey mixed with corn steep liquor, hydrolyzed potato broth, and corn steep liquor (0.65 €·kg^−1^, www.alibaba.com) have been used in media formulations for ω-3 compounds production from heterotrophic microalgae (Table 1). However, despite the high content of protein, carbohydrates and minerals existing in these complex substrates, they cannot be directly assimilated by the majority of microorganisms. Nevertheless, these substrates can be made accessible for microorganisms when they are subjected to pre-treatments steps, in order to release the nutrients needed by microbes. For example, Gong et al. [22] utilized a solid state fermentation and fungal autolysis using the fungal strains *Aspergillus oryzae*, *Penicillium oxalicum* and *Neurospora crassa*, to produce rapeseed meal hydrolysate (RMH). Afterwards, RMH was used as growth medium for heterotrophic growth of the microalga *Crypthecodinium cohnii* to produce DHA. Mendes et al. [21] obtained a carob pulp syrup with high content of total reducing sugars by adding distilled water to fragmented carob pulp (2:1). Afterwards, the suspension was pressed and squeezed, the supernatant was centrifuged, and the liquid fraction was acidified to pH 2 in order to promote the sucrose hydrolysis to obtain glucose and fructose.

### 4.2. Culture Mode

From Table 1 it can be seen that the batch and fed-batch regimes have been used to develop heterotrophic microalgae for lipid production. Algal oil fermentation processes should be preferentially carried out in two stages: in the first phase of active growth phase, the nutrient excess conditions allow cell proliferation, keeping the lipid production low as the carbon is directed towards cell division. In the second phase, the lack of a nutrient, often nitrogen, with concomitant carbon continuously supplied to the fermenter, cell growth and division are halted and the energy previously reserved for DNA/RNA and other protein synthesis will be switched for the production of TAGs rich in DHA. Maintaining the carbon excess condition in the broth is essential not only to promote the lipid synthesis, but also to avoid the endogenous utilization of the internal storage lipids [25]. Other lipid stimulation strategies have been applied to heterotrophic microalgae, such as high salinity [26]. However, it should be noted that the stress conditions that induce the microbial lipid synthesis usually yield low microalgal biomass concentrations. Additionally, sometimes these conditions promote the PUFAs oxidative damage, conducting to the lipids peroxidation an undesirable reaction that breaks down these lipids, making them unsuitable for commercial applications. Thus, the development of new strains that simultaneously produce high amounts of lipids and show antioxidant robustness are desired. An adaptive laboratory evolution (ALE) was constructed by Sun et al. [27] to enhance the *Shizochytrium* DHA production. The authors used two different approaches for the ALE: low temperatures and high salinities to improve the antioxidant resistance of the microalga.

### 4.3. Dissolved Oxygen

The production of microbial lipids, particularly ω-3 compounds, requires high levels of dissolved oxygen (DO) in the broth, in order to promote the formation of double bonds in the final ω-3 fatty acids, but there is controversy concerning this issue. The oxygen requirement depends on the microalgae species, and this nutrient has a key role in the initial phase of biomass production [28]. To ensure oxygen-sufficient conditions in microalgae cultures, adequate agitation and/or aeration rates must be used, since these parameters determine the oxygen availability in the broth. However, caution must be taken since some microalgae species, particularly dinoflagellates, are sensitive to shear stress, although different opinions can be found in the literature about the shear sensitivity of *C. cohnii* cells. Wang and co-workers [29] analyzed the shear stress tolerance of different microalgal species (haptophytes, red algae, diatoms and dinoflagellates) and concluded that dinoflagellates were the most sensitive microorganisms.

During microalgal cultivations, the most important parameters responsible for shear stress are turbulence, eddy size and viscosity, which may affect several cell functions and compartments. Indeed, the cell cycle may also be disturbed by shear stress, as previously demonstrated by Yeung et al. [30], in a work where the authors studied the effect of mechanical agitation on the cell cycle progression of synchronized *C. cohnii* cells. The authors found that when the cells were grown at 150 rpm, a high proportion of cells were arrested in G1 phase; however, as the agitation ceased, the cells normally resumed the cell cycle. On the other hand, Hu et al. [31] observed that no cell lysis occurred when *C. cohnii* cells were grown under air flow rates ranging from 0 to 100 mL min^−1^; however, above 50 mL min^−1^, damage on the flagella was observed, resulting in loss of motility. Moreover, when the flow was interrupted, motility was recovered.

Additionally, using flow cytometry in association to propidium iodide staining procedure, Yeung et al. [30] studied the effects of mechanical agitation on cell cycle progression, having observed that mechanical agitation induced transient cell cycle arrest at G1 phase, in both the heterotrophic dinoflagellate *C. cohnii* and the photosynthetic dinoflagellate *Heteroscapsa triquetra*.

Positive effects of agitation on *C. cohnii* 30772 growth and lipid production have also been reported. De Swaaf et al. [17] reported an enhancement in *C. cohnii* optical density readings by 400% in shake flask experiments (50 mL in 250 mL flasks), when the shaking speed was increased from 50 to 100 rpm, and microscopic studies revealed no detrimental effects of 100 rpm on cell physiology in comparison to 50 rpm. Safdar et al. [32] found a slight increase in biomass and DHA production as *C. cohnii* ATCC 30555 was grown from 150 to 450 rpm.

In any case, when producing microalgal lipids through heterotrophic metabolism, the cell oxygen requirements must be carefully considered, particularly during the microalgae active growth phase, being cell damage monitoring desirable, to avoid cell growth disturbance. Guo et al. [33] developed a novel bioreactor design which enables a high oxygen supply, in combination with a DO-control strategy to improve the microalgal DHA production. A porous-membrane-blade impeller was used, leading to enhanced aeration and increased DO, which constitutes a novel bioreactor design. According to the authors, this novel bioreactor can significantly increase the DO concentration compared to a conventional bioreactor, facilitating cell proliferation and lipid accumulation without cell damage.

### 4.4. Culture Medium pH

Culture medium pH affects the microalgal biomass and DHA production. The optimal pH for biomass and lipid production depends on the microalga strain. According to Gong et al. [22] the optimal pH for *C. cohnii* ATCC 30556 growth, lipid and DHA production was 7.2. However, Safdar et al. [34] reported the optimal pH of 6.5 for *C. cohnii* ATCC 30556 growth, lipid and DHA production. Nevertheless, both authors found that pH values lower than 5.0 or higher than 9.0 were unfavorable for *C. cohnii* growth. This is due to the fact that K^+^/proton and Na^+^/proton antiport systems, as well as modulation of primary cellular proton pumps, are responsible for the intracellular pH maintenance, which requires an electrical potential across the cell membrane. In this way, when *C. cohnii* cells are grown at a pH far from the optimal pH, the cells will channel energy to maintain the physiologically normal intracellular pH, which will result in biomass production and specific growth rate decrease.

According to Wu et al. [35], the highest biomass, lipid and DHA production for *Schizochytrium* sp. S31 was achieved near the neutral pH (7.0). At the pH range 5.0–7.0, glucose was completely exhausted but the lipid and DHA production was lower at medium pH lower than 7.0. Above pH 7.0, growth and lipid production did not occur. Therefore, the medium pH should be maintained as close as possible to the optimal required by the particular microalgae, otherwise the organism has to dissipate energy attempting to restore the optimal pH that will not be used for growth and lipid production.

### 4.5. Temperature

Temperature is considered a key factor for algal growth and biomass biochemical composition. It can also be a factor that influences the global process energy consumption. Usually, higher fermentation temperatures increase cell growth, whilst lower temperatures increase production of unsaturated fatty acids. Safdar et al. [32] reported a negative correlation between DHA content and cultivation temperature. Higher DHA content was obtained at the temperature range of 15–20 °C, which was almost 40% higher than that found at 40 °C. Lipid content also decreased with the increase in temperature, reaching its maximum at the temperature of 20 °C, while biomass production attained its maximum at 25–30 °C. This is due to the fact that at low temperature, the cell membrane fluidity decreases and, in order to compensate this and maintain the cellular membrane structure and properties, cells respond by promoting the over-expression of the genes for desaturases (acyl-CoA desaturases, acyl-ACP desaturases, and acyl-lipid desaturases) which results in the desaturation of the membrane lipids, increasing the unsaturated fatty acids production that help to maintain the membrane fluidity [9].

Zhu et al. [20] reported that *Schizochytrium limacinum* could grow at temperatures ranging from 16 °C to 37 °C, while the optimum temperature of DHA production was obtained at 23 °C. It seems that, regardless the microalgae genus, the optimal temperature for microalgae growth does not coincide with the optimal temperature for DHA production.

### 4.6. Salinity

Most of the heterotrophic microalgae which are ω-3 compounds producers are marine microorganisms. Therefore, they need a saline culture medium to grow. Salinity affects the growth of marine microorganisms by controlling the cytoplasmic ion gradient and the activity of enzymes involved in cell wall expansion [36].

Usually for marine microalgae, the salinity levels are adjusted to mimic those found in the sea. The level of salt in the biomass for optimum fermentation varies widely between different microalgae.

De Swaaf et al. [24] reported a minimum sea salt concentration of 17.8 g L^−1^ for optimal growth of *C. cohnii*. However, the seawater salinity is damaging for steel cultivation vessels used in large scale, corroding the walls. The development of a low-chloride medium and the use of adapted strains via classical strain development techniques may overcome this problem [25]. Nonetheless, a patent protects the use of low chloride medium, which limits its use [37]. The ability of microorganisms to grow on low saline medium is considered an important requisite of industrial oleaginous microorganisms [38]. Currently, 304 is the stainless steel standard grade that is used in bioreactors manufacturing, which can endure 0.3–0.5 g L^−1^ NaCl; bioreactor vessels constructed with 316 stainless steel standard grade can withstand up to 1.6 g L^−1^ NaCl, but it is more expensive. Even so, these salt concentrations are too low for the cultivation of seawater organisms, such as *C. cohnii*. Custom-made resistant stainless steel or coatings is a solution, but requires high investment costs [37,39]. Nevertheless, most of the bioreactors used by Martek Biosciences Corporation are made of high-grade stainless steel (types 317L, 2205 or AL6XN), also the used strains accept lower chloride concentrations [25,28]. Thaustochytrids, despite being marine organisms, can be grown in a low salt environment. *Schizochytrium* is high tolerant to salinity and could grow over a wide range of salinity 5–35 ppt which is a significant advantage for commercial production.

### 4.7. Microalgal Culture Monitoring by Flow Cytometry (FC)

Most of the recent works reporting lipid/DHA production from heterotrophic microalgae use traditional methods to monitor the microalgae growth during the culture development, such as optical density, dry cell weight and cell counting [19,22,23,24,25,26,27,28,29,30,31,32,33,34,35,36,37,38,39,40]. However, these techniques provide no information on cell physiological states. This information is important when cells are grown in adverse and harsh conditions, such as media containing industrial effluents/wastes/residues (Table 1) as they also contain inhibitory compounds; in other cases, the feedstock pre-treatment step necessary to release monomeric sugars to be converted by microorganisms may also release inhibitory compounds that may induce cell death or damage that affect its metabolism, which will reduce the process performance. An example of the inhibitory effect on microorganisms of a waste containing toxic compounds was reported by Sarma et al. [41] who studied the effect of the glycerol impurities, i.e., methanol, NaCl and soaps on the hydrogen producer *Enterobacter aerogenes* bacterium, and concluded that the soaps and methanol have a significant inhibitory effect on the microbial hydrogen production.

FC coupled with specific dyes gives simultaneous information, near real time (at-line), on several cell functions and compartments, providing information on cellular stress response and allows understanding the cell survival mechanisms that are triggered by these conditions. The understanding of such mechanisms will allow the development of more tolerant microalgal strains to these inhibitors and the use of more efficient bioprocess control strategies based on the at-line multi-parameter cytometric information.

Microalgae cells are ideal for flow cytometric analysis since they are unicellular and larger than most of the microbes, being easily differentiated from the background and noise. Lopes da Silva et al. [42] used FC coupled with propidium iodide to monitor the cell membrane integrity of *C. cohnii* CCMP 316 cells in the presence of increasing concentrations of n-dodecane, which was used as an oxygen vector, in order to improve DHA production. Nonetheless, this technique has seldom been used to microalgal lipid production.

Traditional techniques for the quantification and characterization of lipids in microalgae cells rely on time-consuming, labor- and equipment- intensive methods such as the Gas chromatography -mass spectrometry (GC-MS) analysis of fatty acid methyl esters (FAME). The quantification of cellular lipids is generally performed through gravimetric lipid detection methods. These methods are time-consuming and show several disadvantages such as the need of high amounts for toxic organic solvents and significant amounts of biomass for lipid quantification. Importantly, the gravimetric methods usually last several days and, frequently, the results are only available when the process is over, being impossible to change the cultivation conditions during the process development. By contrast, FC can at-line monitor the microbial lipid production, with results becoming available a few minutes after the sample collection. With this information, obtained near real time, it is possible to change the operational conditions (such as carbon to nitrogen ratio, feed and aeration rates, etc.) during the cultivation evolution, in order to enhance the cell lipid production. The lipid production of *C. cohnii* has been quantified by FC using the fluorochrome Nile Red [43]. When monitoring the microalgal lipid production, it is also important to monitor cell viability, as a high proportion of dead cells present in any part of the bioprocess will be detrimental, decreasing the process yield [44].

## 5. Downstream Processing

### 5.1. Oil Extraction

**Figure 2:**
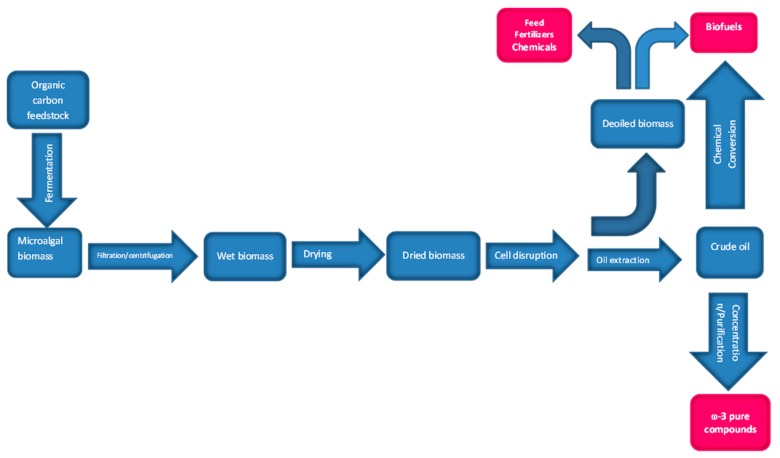
Process flow diagram of a heterotrophic microalgal biorefinery.

At the end of the microalgae fermentation, biomass must be separated from the broth (Figure 2). This is usually carried out by centrifugation or using a rotary vacuum filtration, or direct filtration [45]. Finally, the spent supernatant can be used to produce biogas [46] in order to avoid an extra processing step for its safe discharge in the water system according to the local environmental regulation.

Oil extraction from the microalgal biomass includes mechanical pressing, homogenization, milling and solvent extraction. A drying step is usually performed in order to produce stable biomass free from water that can be stored for long time periods without deterioration. Usually a spray or drum drying are used. Special attention must be paid to the heat-sensitive intracellular TAGs, avoiding exposure of the biomass to high temperatures, as these compounds may degrade at temperatures above 50 °C.

The next stage involves the disruption of the algal cells to release the oil from the cells. A variety of methods can be used to disrupt the microalgae cells such as high-pressure, homogenization, hydrodynamic cavitation, ultrasonic/microwave/pulsed electronic field treatment, solvent extraction, ionic liquids, surfactants, direct saponification, hydrolytic enzymes and algicidal treatments followed by extraction with solvent [47]. The most used method is solvent extraction, being the common solvents chloroform–methanol, hexane, hexane–isopropanol or other solvent mixtures slightly soluble in each other [48]. Depending on the polarity and/or solubility of the lipid fraction, the adequate solvent or mixture must be chosen for the extraction [15]. However, it should be noted that microbial lipids intended to be used in food industry cannot be extracted with toxic solvents, in order to prevent solvent residues in food. The most cost effective procedure is still extraction with hexane, particularly when the extracted lipids are intended to be used in feed/food/pharmaceutical/nutraceutical applications. In this case, it is necessary to ensure that no residual solvent remains in the oil. A further alternative is the use of supercritical fluid extraction (usually with supercritical CO_2_) that usually does not leave solvent residues in the extracted oil, although this technique is more expensive than the classic solvent extraction methods.

However, the micro-algal oil production process must be carried out with caution, due to the high sensitivity to degradation of the long-chain polyunsaturated fatty acids. The natural protection to the long PUFAs in vivo, due to the anti-oxidants that exist inside the cell, no longer exist once the cell is ruptured. If PUFAs react with oxidized radicals, an inexorable chain reaction will start, leading to rancid and highly odorous oils which become non-edible. Thus, all materials that can trigger the oxidation reaction (e.g., copper, ferrous metal) should be removed from the areas where the extraction and oil storage will be carried out. Due to this, it is also important to avoid cell lysis prior to the drying step. The crude algal oil should be kept cool, usually under a nitrogen environment, before the refining step. Waste or oxidized oil can be diverted to biofuel production. The spent de-oiled biomass may have various applications (Figure 2).

### 5.2. ω-3 Compounds Purification

Currently, methods such as winterization, urea adduction, fractional (molecular) distillation and CO_2_ supercritical fluid extraction are used for the extraction and purification of ω-3 PUFAs from microalgae, at the bench scale.

Winterization is one of the simplest methods employed for concentration of omega-3 fatty acids. This process takes advantage of the existing differences in the melting points of different fatty acids as neat oils or in different solvent systems. The melting points of fatty acids are dependent on their degree of unsaturation. The more saturated fatty acids have higher melting points and crystallize out of the mixtures, leaving behind the more unsaturated fatty acids [49].

Another efficient, simpler and cheaper technique for concentration and purification of ω-3 fatty acids from natural sources is the urea adduction method. The formation of complexes between urea and straight-chain saturated fatty acids promotes an efficient separation for fractionation of free fatty acids or esters [50]. Initially, the TAGs of oil are hydrolyzed into their constituent fatty acids using an alkaline hydrolysis with alcoholic KOH or NaOH. The resultant free fatty acids (FFAs) are then mixed with an ethanolic solution of urea for complex formation. Urea molecules readily form complexes with saturated and monounsaturated fatty acids and crystallize out as a solid phase on cooling that is removed by filtration. The ω-3 fatty acids remain in the liquid fraction. Temperatures from ambient to −20 °C can be used in the crystallization step. The process is considered eco-friendly as it involves environmentally friendly chemicals (FFAs, urea, ethanol, water), considered by the US Food and Drug Administration as safe (Generally Recognized As Safe, GRAS). Low-temperature fractional crystallization, enzyme-catalyzed methods, and molecular distillation can be used sequentially before or after the urea adduction method, resulting in highly purified omega-3 fractions. Mendes et al. [21] developed a simple and inexpensive protocol for DHA concentration and purification from *C. cohnii* biomass, involving sequential saponification and methylation steps in wet biomass, followed by winterization and urea complexation. The most concentrated DHA fraction (99.2% of total fatty acids, TFAs) was obtained for an urea/fatty acid ratio of 3.5 and crystallization temperatures of 4 and 8 °C. The highest DHA recovery (49.9%) was observed for an urea/fatty acid ratio of 4.0 and a crystallization temperature of 24 °C, which corresponds to 89.4% DHA of TFAs.

A ω-3 compounds′ concentrate fraction from the marine dinoflagellate *Scrippsiella* sp. was obtained using a preparative reversed-phase high-performance liquid chromatography method with gradient elution using acetonitrile–chloroform and evaporative light-scattering detection [51].

Lipase enzymatic reactions may be used to enrich PUFAs in oils and to produce different forms and compositions of PUFAs in triglycerides, phospholipids, other fatty acid esters and free fatty acids [38].

Supercritical carbon dioxide extraction is considered a suitable method for PUFAs extraction from microalgae since it uses mild conditions such as low pressures and temperatures, and no toxic solvents are used [52]. Couto et al. [53] used supercritical fluid extraction to concentrate DHA in *C. cohnii* oil. The optimum extraction conditions were found to be 30.0 MPa and 323 K. Under those conditions, the DHA composition attained 72% *w/w* of total fatty acids (TFAs).

However, despite their advantages, the supercritical fluid extraction and the urea complex method have not yet been applied to ω-3 compounds purification at a commercial scale.

The petition of Market Biosciences Corporation (2010, https://www.ams.usda.gov/sites/default/files/media/DHA%20Algal%20Oil%20Petition.pdf) describes a large-scale recovery and purification process of DHA from algae oil produced from *Schizochytrium* sp. and *C. cohnii*. A protease enzyme is used to break a *Schizochytrium* sp. cell wall, in order to release the oil to the broth. As *C. cohnii* has a much more complex cell wall structure, and contains a cellulosic thecal layer, it is not possible to use a protease enzyme to break its wall. Hexane is used to extract *C. cohnii* microalgal oil from dry biomass after high pressure homogenization. Afterwards, the cell debris are removed and the oil is recovered by evaporation.

After extraction, the PUFAs fraction is still not suitable for human consumption due to the presence of impurities, odor, taste, and cloudy appearance. A refining step is required to remove phospholipids, unsaponifiable materials, particulate material and chemical contaminants such as free fatty acids, phosphatides (i.e., lecithin), pigments (i.e., carotenoids, chlorophyll), trace metals, sterols (i.e., cholesterol), mono acyl and diacyl glycerides, waxes, oxidation products and trace contaminants, improving color, clarity and odor.

## 6. EPA/DHA Industrial Production and Applications

The global EPA/DHA market is growing fast since 2013. At this time, the market was estimated to be 124 thousand tonnes and worth almost 2 billion €. It is predicted to be 241 thousand tonnes valued at 4.2 billion € by 2020 [6]. The rising penetration of ω-3 compounds in the Active Pharmaceutical Ingredient (API) market has been the driving force to the industry, triggered by the increasing awareness about the benefits of ω-3 compounds on human health. In addition, recent regulations are favoring the product usage in infant formulations, as one of the driving forces for expanding the range of applications.

Algae oils from *Crypthecodinium cohnii*, *Schizochytrium* sp. and *Ulkenia* sp. are used for enriching food and feed, or as nutritional supplements under different trade names, around the world. The microalgae oil can also be directly used as animal feed ingredients to produce eggs, chicken, and pork meat enriched in DHA. In aquaculture, microalgae are used as a fresh product or as dry pellets that preserve the nutritional content of microalgae. In this case, microalgal biomass is first filtered, after being subjected to dissolved air flotation, flocculation or sedimentation, and then dried to form pellets or directly administrated to livestock [54].

Although the main applications of the microalgal oils were initially in infant nutrition, a rapid development of the oils for adult consumption is currently underway. Infant formula applications represent the most important end application for DHA oil (about 49% of the volume in 2012), followed by dietary supplements (28%), food and beverage (19%) and animal feed (about 4%) [6].

## 7. EPA/DHA Industrial Producers

The DSM enterprise (which bought Martek Biosciences Corporation in 2010) is the major worldwide producer of DHA from algae. It produces algal oils from heterotrophic microorganism *Schizochytrium* sp., such as Life′sDHA™ and Life′s™ OMEGA products. The algae oil contains 50% EPA/DHA (https://www.dsm.com/markets/human-nutrition/en/products/nutritional-lipids.html).

DHASCO oil, for infants, is produced by DSM Nutritional Products, which uses the microalgae *Crypthecodinium cohnii*, presenting a DHA content of 40–45%  *w/w*, and no EPA. Life′s DHA oil, an algal oil for the food, beverage and supplement industries, is also produced by DSM, and is obtained from the microalgae *Schizochytrium* containing 35% or 40% DHA, and low levels of EPA (<2%). Life′s ω-3, a commercial product similar to fish oils in terms of ω-3 composition, contains a minimum of 40% of total DHA and EPA (at least 24% DHA and 12% EPA) and is produced from a *Schizochytrium* strain by DSM.

Lonza, like DSM, is a nutraceutical manufacturer, and sells an oil and powder ingredient, DHAid, for food industry, produced from heterotrophic microalgae. DHAid is a product composed of TAGs (>95%) showing a total DHA content of 38–50%, derived from *Ulkenia* sp.

As mentioned above, the whole microalgal biomass is sold in the market. Solazyme Bunge Renewable Oils (SB oils) produces AlgaPrime DHA, a whole algae product directed for the aquaculture feed market. The facility is based in Brazil and uses sugarcane to grow the microalga *Schizochytrium* producing DHA rich oil. The sugarcane waste is a renewable source of energy for the facility.

## 8. The Heterotrophic Biorefinery Platform

The possibility to obtain multiple products from microalgae (e.g., oils, pigments, proteins and carbohydrates) has led to microalgae-based biorefineries research and development, in order to obtain the widest range of microalgal biomass-derived bioproducts as possible, taking advantage of the various products synthesized by the microalgae, maximizing the value derived from the whole process, with a desired minimal environmental impact (Figure 2).

In the biorefinery concept, still rarely applied to heterotrophic microalgal biomass (see Section 9), ω-3 fatty acids can be separated from the remaining microalgal lipids, which can be used for bioenergy or biodiesel production. For example, due to their high oil productivity, Thraustochytrids have the potential for co-production of both ω-3 PUFAs rich oils, as well as shorter chain fatty acids which are less unsaturated, therefore suitable for biodiesel [55]. Later, Chang et al. [56] isolated an Australian *Aurantiochytrium* sp. strain, TC 20, for co-producing biodiesel and high-value ω-3 PUFAs. The simple fatty acid profile of this strain, having both saturated fatty acids (45–52% *w/w* TFAs as 16:0) and ω-3 PUFAs (39–48% *w/w* TFAs as DHA) as major constituents, make it potentially a very good candidate to co-produce both ω-3 PUFAs and biodiesel. The authors did not separate these lipidic fractions, but suggested a further winterization step for separation of ω-3 PUFAs fraction from the remaining fatty acids fraction that could be used for biodiesel purposes. This procedure has been used to valorize fish canning industry byproducts, producing ω-3 compounds and biodiesel [2].

Different industries are able to use different algal products: the entire microalgal biomass can be used by food, feed and agriculture industries; the pharmaceutical and nutraceutical industries use microalgal high value added lipidic products such as ω-3 PUFAs and carotenoids; the transport industry can use fatty acids derived from the microalgal TAGs for biodiesel; the chemical industry can use products such as glycerol derived from the biodiesel industry; and the deoiled biomass leftovers, rich in carbohydrates, proteins, and minerals, can be used as feed, fertilizer, and substrate for the production of bio-methane. Further, thermo-chemical conversion of this spent biomass may generate fuels and other chemicals (Figure 2). The lowest valued application of the deoiled microalgal biomass may be used for adsorption of dyes and heavy metals from industrial effluents [57].

All these microalgal products, obtained within a biorefinery frame based on the circular bioeconomy principles that aim at zero residues generation may add greater value to the production process pipeline, with improved process economics, and may address four major areas of importance in human society: human health, transportable energy, food and environment security [54].

## 9. Sustainability Assessment of Benchmark—Omega 3 (ω-3) Biorefinery

Most health recommendations indicate a daily consumption of 250–1000 mg of ω-3 fatty acids (EPA and DHA) per adult, which means, for 7 billion people, an annual consumption of about 1.3 million tonnes [5]. These ω-3 fatty acids for nutrition and pharmaceutical applications are usually obtained from fish oil. As referred above, the fish industry, even considering wild fish and aquaculture, will not be enough to satisfy the fish oil demand and a further increase would risk destroying fish populations.

As already stated, ω-3 compounds derived from microalgae oils is currently more expensive than from fish oils, and a possible strategy to make these compounds obtained from microalgae more competitive consists of valorizing all the microalgae biomass fractions, close to the biorefinery concept. There are no reports on such an approach applied to heterotrophic microalgae at a commercial scale. Even at lab-scale, there are no works considering heterotrophic microalgae biomass biorefineries and its sustainability evaluation. For example, a recent research refers the use of aquaculture wastewater as a nutrient substrate for cultivation of microalgae to produce lipids and proteins [58] but neither scaled-up the process, nor evaluated the sustainability of the purposed biorefinery.

A brief description on ω-3 compounds production within two modeled biorefineries will be presented below. For this purpose, we considered that “Benchmark” processes cover the existing simulations in literature or real biorefineries existing in the market. The sustainability indicators considered were cost (utility cost, equipment cost, capital expenditure-CAPEX, operational expenditure-OPEX, direct energy needs, thermal and electric, and derived CO_2_eq emissions).

The reference system concerns the conversion of fish wastes to EPA/DHA explored within a biorefinery concept from a trout processing plant located in Trentino Province, Italy [59] (Table 2). The biorefinery includes the following processes: oil extraction from fish waste, fish oil transesterification with ethanol, and ω-3 concentration based on supercritical CO_2_ fractionation. Proteins are valorized as fishmeal, while glycerol is considered to be a commercial product and, saturated fatty acids and short-chain unsaturated fatty acids are considered to be valorized as liquid biofuel. 870 tonnes/year^−1^ of fish waste are converted throughout a “virtual biorefinery” in 26.6 tonnes/year^−1^ of ω-3 rich oil, 160 tonnes/year^−1^ of fish proteins, and 160 tonnes/year of liquid biofuel. The biofuel, fed to a 100 kW combined heat and power (CHP) unit, allows to produce 720 MWh/year^−1^ of electricity and 870 MWh/year of heat. This CHP unit was sized to cover the total electricity consumption of the plant and provide more than 45% of the thermal energy needs. The sustainability of the processes is quantified in terms of CO_2eq_ emissions from the thermal energy needs (201 g CO_2_ kW^−1^) and from the electricity needs, Italian mix (352 g kWh^−1^).

**Figure 3:**
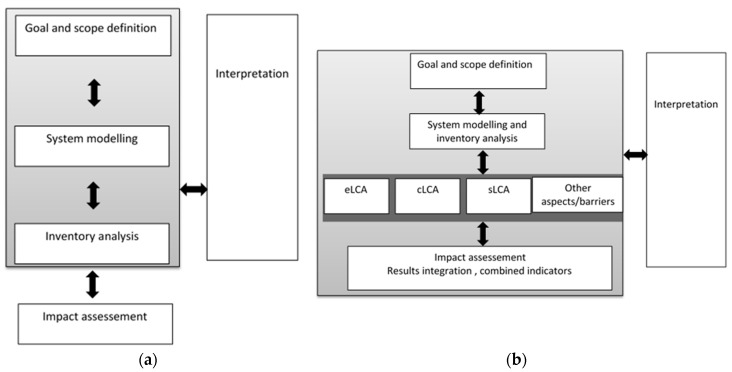
Differences in Environmental life cycle analysis eLCA (**a**) and Integrated life cycle sustainability assessment (ILCSA) (**b**) methodologies (adapted from PUFAChain Project [6], and Keller et al., 2015 [61].

Another modeled biorefinery example concerns the European project PUFACHAIN [60]. This project evaluated the potential of autotrophic microalgae in Europe in a photosynthetic area of 10 ha to 100 ha, in Lisbon, Munich and Oslo (Table 2). The methodology used for the sustainability analysis was the Integrated Life Cycle Sustainability Assessment (ILCSA) that evaluates the environmental impacts, the costs and social impacts of the process [61] (Figure 3). This methodology has also been used in the EC-funded FP7 projects GLYFINERY (GA 571 No. 213506), BIOCORE (GA No. 241566), SUPRABIO (GA No. 241640), BIOLYFE (GA No. 572 239204), SWEETFUEL (GA No. 227422), OPTIMA (GA No. 289642) and D-FACTORY (GA No. 573 613870). The microalgae cultivation processing (in closed system unilayer horizontal tubular photobioreactors, energy requirements provided by solar panels) uses electricity from the grid and heat from natural gas boilers (harvesting by membrane concentration, recycling of waste water, spray drying, supercritical CO_2_ extraction, oil concentration), and final products (PUFAs, extraction cake, removed fatty acids and glycerol) use are considered, including land use effects. The reference system for comparison is the fermentation of fungi/protists/microalgae-like, co-processed with wastes of fish cuttings for PUFAs, crude oil for glycerol, soy cultivation for extraction cake and rapeseed cultivation for removed fatty acids. The reference system emits much less CO_2eq_ than the purposed autotrophic paths, even with solar power incorporation.

Table 2 summarizes our findings from the reviewed literature. The examples here have different boundaries regarding costs and CO_2eq_ emissions, so the comparison between the two is quite difficult. Nevertheless, from these documents it is possible to conclude that autotrophic microalgae seem to need further energy/cost improvements (the microalgae production seems to be the most expensive stage, more than 50% of the total costs). Both examples shown in Table 2 used the supercritical CO_2_ extraction for the microalgal oil extraction, which, although is a suitable method for PUFAs extracting (as referred in Section 5), can be a major energy consumer and, therefore, a major CO_2eq_ contributor. In the examples shown in Table 2, this extraction method requires ~50% of thermal and ~60% of electricity needs.

**Figure 4:**
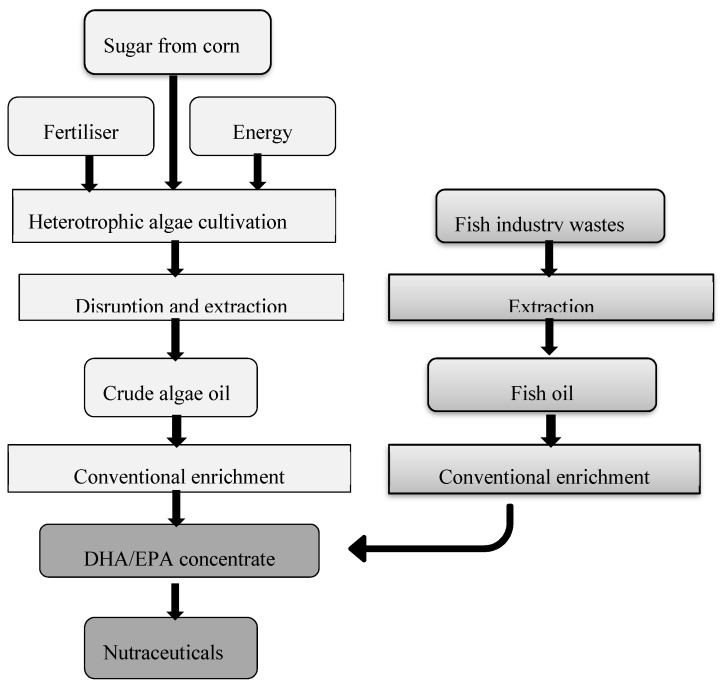
Reference future industrial PUFA production as the future vision reported in the European project PUFACHAIN: heterotrophic algae co-processed with fish industry wastes [6].

The industrial reference system that is described in the PUFACHAIN project is a future scenery vision of what could be the most sustainable system for ω-3 compounds production: mixing the heterotrophic microalgal oils with fish industry waste oils to obtain a common ω-3 compound concentrate fraction (Figure 4). However, as commented before, fish oils present an unpleasant odor and smell, and may be contaminated by polychlorobiphenyls (PCBs) and heavy metals, making them inappropriate to be incorporated in foods, namely in infant formulas, or to be used in pharmaceutical formulations, unless expensive concentration/purification steps are used. Therefore, their mixing with pure microalgal oils would sacrifice the benefits that make the pure microalgal oils so attractive to the consumers, such as their high proportion in DHA/EPA (which makes easier their concentration/purification), the absence of ocean-borne contaminants, and their vegetarian nature, a source currently very popular and searched, particularly by young people. Furthermore, as marine fish oils contain a complex mixture of fatty acids with varying lengths and degrees of unsaturation, its mixing with pure microalgal oils would require additional expensive DHA purification steps, before application. Therefore, a future vision of a dedicated microalgae biorefinery should be critically equated and evaluated. Indeed, the inexistence of research on a dedicated heterotrophic microalgae biorefinery integrated with an LCA or ILCSA analysis represents the opportunity to explore this issue in future research. Of course, the CO_2_ biogenic emissions produced by heterotrophic microalgae must be included in these future LCA or ILCSA studies. Despite the difficulty in finding this information from literature, it can be calculated based on the knowledge of the growth mechanism: carbon (C) mass incorporated into new biomass (CO_2_/C) equaled 0.4–1.4 g·CO_2_/g·C [13], for a substrate provision of 5–60 glucose/L, and the dry cell yield: average 9 g/L [3].

## 10. Bottlenecks, Challenges and Future Perspectives

The development of heterotrophic microalgae processes towards ω-3 compounds may provide an alternative biotechnological way to produce useful products that would otherwise be fully and unsustainably produced from other living resources such as marine fatty fish, which show several drawbacks, as above referred.

However, such an approach still presents several challenges and hurdles that must be solved to enhance ω-compounds heterotrophic microalgae production.

Since bacteria usually grow faster than microalgae, bacterial contaminations are very common in heterotrophic microalgal cultures, becoming the major (prevailing) population in a short time period. This will contaminate the microalgal biomass, which will be inadequate for commercial applications. Therefore, a previous sterilization step of the medium culture and equipment is required before the inoculation, which is a high energy demanding step, requiring expensive equipment such as autoclaves, laminar flow cabinets and boilers, which increases the global process costs, particularly at large scale. This is particularly critical if using industrial wastes/effluents/residues in the medium culture that often contains high microbial load. Therefore, low cost sterilization methods such as the use of sodium hypochlorite [62] that may replace the expensive sterilization methods at large scale should be investigated, in order to reduce this cost.

Another major issue regarding the heterotrophic microalgae cultures concerns the need of an efficient mixing and aeration in the broth, to avoid mass transfer limitations which reduce the process yield. Mixing times and aeration rates from lab/bench bioreactors to large-scale production facilities increase dramatically, affecting the overall bioreactor performance. If there is an inefficient mixing in the vessel, spatial heterogeneities in nutrient concentration will originate stagnant regions that results in cellular stress, which will negatively affect the overall process productivity. This is particularly critical when using obligatory aerobic microalgae at a large scale, since their oxygen requirements are high and often the microbial cells are exposed to environments under limited oxygen conditions [2], but always bearing in mind that these microorganisms are particularly vulnerable to shear stress. In this sense, heterotrophic microalgae genetic engineering could play an important role, being a way to develop more robust strains to stressful conditions. This improvement must be carried out towards biological development of more robust strains, and technological development of bioreactors able to provide enough oxygen under gentle stirring without the presence of dead zones.

The extraction and purification methods for microalgal PUFAs have been studied mainly at laboratory level. However, large-scale intracellular metabolites recovery is still incipient, since not all the methods of cell disruption, extraction or purification are scalable. Moreover, these methods are high energy demanding. Nonetheless, some technologies like bead mill, high-pressure homogenization, winterization and urea adducts can be viable at a large scale, which needs to be demonstrated.

The use of heterotrophic microalgae biorefineries in which heterotrophic microalgae treat effluents and produce a range of bioproducts such as PUFAs, and biofuels, is based on the circular economy principles, and it is indispensable to achieve an environmental and economical sustainable process. Indeed, the full application of a real circular bioeconomy in this area is desired in the near future, taking advantage of all the heterotrophic microalgal biomass fractions, exploring the integration of new efficient technologies for extraction, concentration, fractionation, conversion and purification of lipids from microalgae, and highlighting the need for recycling the side-streams and wastes generated in the whole process, driving circular excellence. On the other hand, the possibility of co-processing heterotrophic microalgae and fish wastes for ω-3 compounds production could boost circular economy and should also be considered in future research studies.

At last, but not the least, there is an urgent need for evaluating heterotrophic microalgae biorefineries sustainability. In terms of sustainability indicators, an integrated life cycle sustainability analysis methodology (combining economic, environmental and social life cycles) is desirable considering heterotrophic microalgae alone, or combined with fish-wastes as feedstock obtain ω-3 compounds. Less than 26 tonne (tonne CO_2eq_ PUFA)^−1^ (Table 2), regarding energy consumption could be the goal for the environmental performance and 400 € (tonne PUFA)^−1^ for the economic costs.

## Figures and Tables

**Table 1:** Heterotrophic microalgae and microalgae-like strains that produce ω-3 compounds, as well as the carbon sources that have been used in the media formulations.

Substrate	Feedstock	Microorganism	Cultivation Mode/Time	Lipid Production	EPA/DHA Production	Reference
Pure sources	Glucose	*C. cohnii* ATCC 30772	2 L bioreactor, batch/91 h	3.79 g·L^−1^	1.6 g·L^−1^ DHA	[16]
	Ethanol	*C. cohnii* ATCC 30772	2 L bioreactor, fed-batch/220 h	42.2% w·w^−1^, 35 g·L^−1^	11.7 g·L^−1^ DHA	[17]
	Acetate	*C. cohnii* ATCC 30772	2 L bioreactor, fed-batch/120 h	61.0 g·L^−1^, 56.0% w·w^−1^	19 g·L^−1^ DHA	[17]
	Glycerol	*C. cohnii* CCMP 316	2 L stirred tank bioreactor/Batch mode/8 days	2.34 g·L^−1^, 36.5% w·w^−1^	DHA: 49 mg·g^−1^	[18]
Food industry effluents/wastes	Food waste hydrolysate	*Schizochytrium mangrovei Chlorella pyrenoidosa*	2 L bioreactorBatch mode/7 days	3.30 g·L^−1^; 16.49% w·w^−1^ 1.05 g·L^−1^; 20.99% w·w^−1^	85.5 ± 11.2 mg·g^−1^ DHA	[19]
	Sweet sorghum juice	*Schizochytrium limacinum*	250 mL flasksBatch mode/5 days	6.90 g·L^−1^; 73.4% w·w^−1^	273 mg·g^−1^ DHA1.1 mg·g^−1^ EPA	[20]
	Carob pulp syrup	*C. cohnii* CCMP 316	2 L bioreactor, fed-batch	9.2% w·w^−1^ (as TFA)	1.99 g·L^−1^ DHA45.2 mg·g^−1^ DHA	[21]
	Rapeseed meal hydrolysate + crude waste molasses	*C. cohnii* ATCC 30772	500 mL-Erlenmeyers, batch/7 days	27.3% w·w^−1^, 26.9 mg·L^−1^	8.72 mg·L^−1^ DHA22–34 % w·w^−1^ DHA of TFA	[22]
	Cheese whey + Corn steep liquor	*Crypthecodinium cohnii* CCMP 316	250 mL-Erlenmeyers	28.7% w·w^−1^	8.5–27% w·w^−1^ DHA of TFA	[18]

**Table 2:** Sustainability indicators for ω-3 compounds (EPA and DHA) biorefinery.

Biomass	Biorefinery	Processes	Products	Cost	Energy	CO2eq	Reference
Fish wastes 871 tonne year^−1^	Modeled Aspen Plus™	oil extraction from fish waste; fish oil trans-esterification with ethanol, and supercritical CO_2_ fractionation	Proteins for fishmeal (160 tonne year^−1^)’; Biofuel (160 tonne (year for CHP^−1^)); ω-3 concentrates for the nutraceutical sector (26.64 tonne year^−1^ or 30 kg PUFA (kg dry microalgae)^−1^; PUFA (58% mass fraction in EPA and DHA)	3.34 M€-Equipment 178 k€ (year utility)^−1^	Electricity needs 716 MWh year^−1^ (100% from biofuel CHP); Heat 1919 MWh year^−1^ (45% from CHP)	695 tonne year^−1^; Or 26 tonne (tonne PUFA)^−1^	[59]
Phototrophic algae (*Prorocentrum cassubicum*, *Thalassiosira weissflogii* and a combination of *Chloridella simplex* and *Raphidonema nivale Lagerheim*) 390–4900 ton dry weight year^−1^	Modeled industrial scale 10–100 ha of land use	algae production; algae harvesting; cell disruption and spray drying and supercritical CO_2_-extraction and oil processing	5–152 tonne PUFA year^−1^ or 5–9 g PUFA kg dry microalgae^−1^; extracted cake 21–3800 tonne year^−1^; oil wastes 8–200 tonne year^−1^	Capital cost as CAPEX ^a^ 2.6–41.4 M€ year^−1^; Operational cost as OPEX ^b^ 2–31 M€ year^−1^; 400–1500 € (kg PUFA)^−1^	Electricity 1000–95,000 MWh year^−1^; Heat 80,000–15,000,000 MJ year^−1^; or 22–4200 MWh year^−1^	1750 tonne (tonne PUFA)^−1^ or 350 tonne (tonne PUFA)^−1^ if more solar power is considered; Or 9–16 tonne (tonne autotrophic)^−1^; Microalgae^−1^	[6]

^a^ includes Offices, warehouse and workshop-Laboratories-Control and electrical systems-Civil engineering-Licencing, Engineering, procurement and construction (EPC) and contractor costs-Water treatment systems-Nutritive medium preparation systems-Production systems-Thermo-regulation system-Effluents and medium recycling; 10 year depreciation time for equipment; ^b^ includes Nutrients, CO_2_, Water (20–29% of the costs), Silicates, Salt, Electricity, Waste, O & M costs, Labour costs (33–77% of the costs).
